# Physicians in Greece’s Emergency Departments: Attitudes, Readiness, and Need for Formal Training

**DOI:** 10.5811/westjem.39964

**Published:** 2025-07-09

**Authors:** Sarah Aly, Dimitrious Babales, Olympia Kouliou, Andrew Ulrich, Eleanor Reid, Dimitrios Tsiftsis

**Affiliations:** * Yale University School of Medicine, Department of Emergency Medicine, New Haven, Connecticut; † Larissa General Hospital, Department of Emergency Medicine, Larissa, Greece; ‡ Larissa General Hospital, Department of Anesthesiology, Larissa, Greece; § Nikaia General Hospital, Department of Emergency Medicine, Nikaia, Greece

## Abstract

**Introduction:**

Despite the recent recognition of emergency medicine (EM) as a distinct specialty in Greece, emergency departments(ED) there continue to be staffed by physicians with training in other medical specialties, although some hold EM certifications. In this study we aimed to evaluate the perceived level of competency and preparedness of physicians who work in EDs in Greece. We also sought to identify gaps in clinical EM expertise, solicit opinions on the need for EM residency training in Greece, and determine the well-being and job satisfaction of physicians practicing in Greek EDs.

**Methods:**

This was a mixed-methods, cross-sectional, electronic, nationally representative survey of physicians working in EDs across all health districts in Greece. The survey was administered in Greek and anonymously conducted online. We used the Pearson chi-squared test to determine whether there was an association between EM certification and comfort with seeing subsets of patients. The study received institutional review board approval, and all participants signed an online consent form.

**Results:**

The study surveyed 105 of 263 physicians working in 52 Greek EDs (39.9% response rate). We found that of the 105 physicians surveyed, 63 (60.0%) were not certified in EM. A Pearson chi-squared test revealed a significant association between comfort level in seeing pediatric, trauma, and critically ill patients, and EM certification (X^2^ = 13.37, *P* = .001). Qualitative analysis found that physicians had a desire to engage in training opportunities, with many citing cost, time, and age as barriers. Despite these challenges, 64.1% of physicians reported satisfaction with their decision to work in the ED.

**Conclusion:**

Most frontline emergency physicians working in Greece are uncomfortable caring for the full breadth of ED patients. This survey represents the first assessment of the attitudes, clinical preparedness, and perceived need for EM residency training among emergency physicians in Greece. Critical next steps should include enhanced training on targeted aspects of emergency care for practicing emergency physicians in the nation and continued efforts to establish formal EM residency training in Greece.

## INTRODUCTION

Greece is a high-income country located in southeastern Europe divided into seven health regions. It boasts a centralized National Health Care System, *Eθνικό ∑ύστημα Yγɛίας* (ESY), regulated by the Ministry of Health (MoH), which is responsible for medical staffing and funding of all public health facilities including 52 emergency departments (ED) across the country.^1^,^2^,^3^ Since 2000, emergency medicine (EM) has become increasingly more important in Greece as the country has experienced an increased number of climate change-related natural disasters causing mass casualty events, including wildfires, floods, earthquakes, volcanic eruptions, and tsunamis.^4^

**Figure 1:**
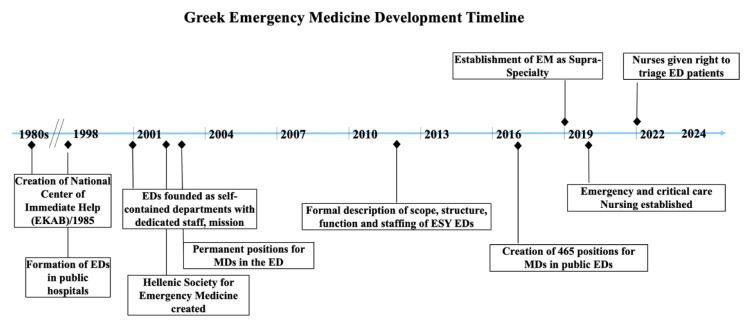
Timeline of the development of emergency medicine in Greece. *EM*, emergency medicine; *ED*, emergency department; *EKAB*, National EMS Services; *ESY*, Eθνικό ∑ύστημα Yγɛίας; *MD*, physician.

While Greece has a long history in medicine, EM as a specialty has been slow to develop.^5^ Emergency departments were created in all Greek public hospitals in 1998, using the specialty-based model (eg, cardiologists and gynecologists see their respective patients in specialty-allocated beds in the ED). In early 2000, every ED was allocated 1–3 permanent positions for physicians who had trained in one of the four major specialties: surgery, general medicine, internal medicine, or cardiology. Unfortunately, very few physicians applied, as the role was poorly defined, and EM as a specialty was largely unknown. In 2017, to strengthen emergency care capacity in Greece, an additional 465 permanent ED positions were announced. In 2019, the MoH first recognized EM as a “superspecialty,” to be awarded after two years of formal training beyond residency (Figure 1). An alternative second pathway to the title of specialist in EM was through grandfathering in, which was awarded to those who had practiced in an ED for five years prior to the creation of the superspecialty.

In 2020, Greece was recovering from an austerity economic program imposed by the European Union and International Monetary Fund, whereby all public sector expenses were under tight control. Thus, very few placements for ESY physicians were made available, creating interest in the available ED positions as physicians sought a way to join ESY. However, most applicants had no direct exposure to or training in EM, thus preferring to practice within the allocated beds of their specialty within the ED.

The COVID-19 pandemic shifted the focus from developing EDs and ward-based internal medicine and intensive care units (ICU) to care for the influx of sick medical patients. Most EM development initiatives, including training and staffing, were put on pause during this time.^6^ In the post-COVID-19 era, the focus in Greece has shifted back to improving emergency care capacity: more EM superspecialty training centers have been created and, more importantly, the culture of EM is growing. Emergency physicians have also been exposed to the full spectrum of EM through the efforts of the Hellenic Society for Emergency Medicine (HeSEM).^7^ The HeSEM was established in 2002 with the goal of advancing the field of EM in Greece. The HeSEM is currently advocating for the establishment of Greece’s first EM residency training program while also enhancing EM training for ED-based physicians who are not EM-certified. The goal is to standardize ED practices and elevate emergency care quality nationwide. International collaborations, political lobbying, certified educational activities, and scientific meetings have helped further the EM cause in Greece.

Despite this, the 52 EDs across Greece are currently staffed with just 263 physicians; however, only a small fraction of these are certified as EM-supraspecialists. Thus, Greek EDs today are largely run by non-EM trained but ED-based physicians from other specialties. The Greek model of emergency care by non-certified EM attendings is not unique in Europe or globally; however it is under-studied, making it imperative to determine whether frontline physicians feel adequately trained to serve the full breadth of emergency patients. It is unclear whether the physicians practicing in Greek EDs feel prepared for or satisfied by the challenges of a clinical career in EM, nor is it clear what their specific needs might be as far as additional training or support. To this end, we conducted an anonymous, electronic survey of physicians practicing in Greek EDs.

**Population Health Research Capsule**

What do we already know about this issue?

*Greek EDs are staffed by non-EM trained doctors. Some are EM certified. Their preparedness, satisfaction, and training needs are unknown*.

What was the research question?

*Do Greek emergency physicians feel comfortable treating patients in the ED, and how do they perceive the need for formal EM training?*

What was the major inding of the study?

*EM-certified doctors felt more comfortable treating pediatrics, trauma and critical care patients than those trained in other specialites (P=0.0096)*.

How does this improve population health?

*Identifying physician comfort and training needs informs policy to improve emergency care and, consequently, patient outcomes in Greek EDs*.

## METHODS

This was a cross-sectional, observational, mixed-methods study (quantitative and qualitative) conducted via electronic survey of physicians practicing in Greek EDs. First, co-authors SA and ER convened an expert committee of medical directors of two major Greek EDs (co-authors DT and DB) who practice clinical EM to discuss the main issues facing physicians practicing in Greek EDs. A de novo survey was then derived, comprised of questions aimed to assess emergency physicians’ training, job satisfaction, perceived competencies in critical areas of EM practice, and perceptions of the need for formal EM training.

The survey was translated from English to Greek and back-translated to English by fluent, bilingual native Greek speakers. The survey was uploaded to an online survey tool (Qualtrics International, Inc, Provo, UT). An initial pilot version of the survey went live and was completed by a subset of participants, who then filled out the final version of the survey. Questions 30–32 were consequently added, and modifications to language and question numbering were made. A link to the final version of the survey was disseminated via email from ED medical directors to their staff on April 3, 2024. Upon following the link, study subjects were prompted to create a unique identifier to decrease the likelihood of duplicate responses and to enable them to return to their survey later to complete it. The study questionnaire began with an informed consent question followed by 30 questions. The survey took approximately 15–25 minutes to complete. The study ran until June 17, 2024. Please see Appendix 1 for the English-language version of the survey. Descriptive analysis was done via Microsoft Excel (Microsoft Corporation, Redmond, WA), with missing responses excluded from the relevant analyses.

We performed a Pearson chi-squared test to assess the association between comfort level and certification status using R (R Foundation for Statistical Computing, Vienna, Austria). We created a contingency table based on the observed frequencies of comfort levels across pediatric, trauma, and critically ill patients and certification status. Standardized residuals were extracted to evaluate the strength of the association between categories with the significant threshold set at |R| > 2. Qualitative data was uploaded for analysis to NVivo 14 (Lumivero LLC, Denver, CO).

## RESULTS

**Figure 2:**
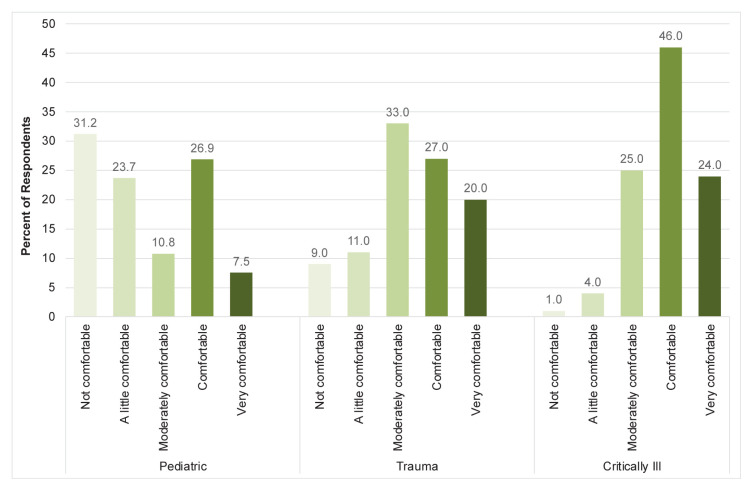
Emergency physician comfort levels when treating patients according to patient type in a study of physician attitudes regarding need for emergency medicine training in Greece.

The survey was distributed to 263 physicians across all 52 EDs in Greece, representing the nation’s seven healthcare administrative regions. There were 171 responses. Of these, 19 were duplicates, 47 surveys had no answers filled out, and two were only partially completed. We excluded from the analysis the 66 duplicates and unanswered surveys. Thus, the response rate was 39.9%. Basic characteristics of the respondents are noted in the Table. Figure 2 shows levels of comfort with pediatric, trauma, and critically ill patients in the ED. We performed a Pearson chi-squared test to examine whether there was a significant difference between levels of comfort and EM certification. Due to inadequate power, we combined pediatric, trauma, and critically ill questions. The chi-squared test revealed a significant association between comfort level and certification status (X^2^ = 13.37, *P*-value = .0096). Standardized residuals were then done to examine which comfort levels contributed most to the test statistic. The “not comfortable” and “very comfortable” categories showed strong, statistically significant deviations (|R|= 2.50 and 2.16, respectively). Specifically, the “not comfortable” category showed a significant over-representation for those who were not EM certified (R = 2.50) and an under-representation for those who were EM certified (R = −2.50). Similarly, the “very comfortable” category exhibited a significant under-representation for those who were not EM certified (R = −2.16) and an over-representation for those who were EM certified (R= 2.16). The remaining answers did not show statistically significant differences.

A total of 95 (92.2%) respondents reported that training in EM is a necessity in Greece. These findings can be contextualized by the fact that 36 (35.0%) respondents reported asking for a consultation from other medical professionals on more than 20% of their patients. “When it is outside the scope of my main specialty” was the most common reason for seeking consultation, making up 34 (33.0%) responses. Notably, a significant proportion of physicians working in Greek EDs also reported feeling overwhelmed, with 20 (19.1%) reporting that they treated more than 4.5 patients per hour.

**Figure 3:**
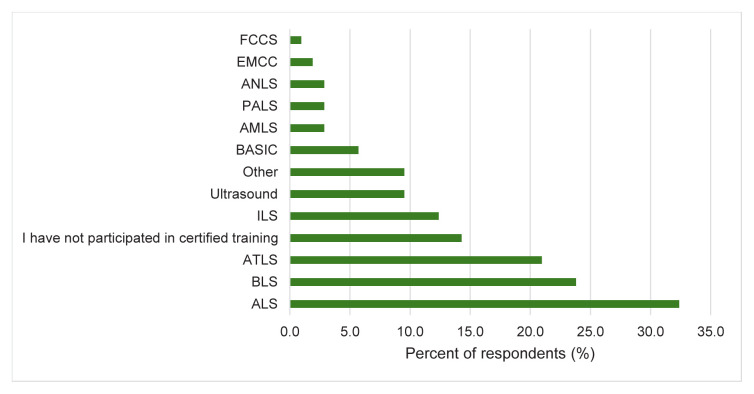
Certifications attained by respondents in a study of physician attitudes regarding need for emergency medicine training in Greece. *FCSS*, Fundamentals of Critical Care Support; *EMCC*, European Master in Critical Care; *ANLS*, Advanced Neonatal Life Support*; PALS*, Pediatric Advanced Life Support; *AMLS*, Advanced Medical Life Support; *BASIC*, Basic Assessment and Support in Intensive Care*; ILS*, Immediate Life Support; *ATLS*, Advanced Trauma Life Support*; BLS*, Basic Life Support*; ALS*, Advanced Life Support.

A minority of respondents attended accredited supplemental training courses, and even fewer were currently accredited in updated training, with lack of time and cost listed as the main factors why these were not attained. Advanced Life Support is the most commonly held certification, with 34 (32.4%) individuals certified. Fundamentals of Critical Care Support and European Master in Critical Care are the least common, with only two (1.9%) and one (1.0%) respondents certified in these courses, respectively. The remaining distribution is highlighted in Figure 3 below. Notably, 30 (28.6%) participants held instructor status in one of these courses. Please see Appendix 2 for a description of each of these certifications.

The qualitative analysis found that many physicians who work in Greek EDs report being unable to attend further training and certification classes due to cost, lack of time, and concern about their ability to learn new skills at an older age. This is despite reporting that they felt they lacked in skills and confidence and needed improved training in critical care, trauma, and pediatrics. The qualitative analysis also found that they had low levels of job satisfaction. This is despite findings within the quantitative analysis that found that 62 (64.1%) physicians reported that they felt they had made a “good choice” in working in the ED, and only 23 (22.3%) physicians reported that they had “made a mistake.” The remaining 14 (13.6%) physicians reported that they still weren’t sure whether it was a good choice to work in the ED.

## DISCUSSION

The creation of EM specialty training is considered necessary by most physicians practicing on the front lines of Greek emergency care. Working in the ED has provided these physicians insight into the Greek EM system, which include deficiencies that could be addressed by formal EM training. Emergency medicine, which was a largely unknown medical specialty in Greece until recently, represents an exciting, much-needed paradigm shift for a system with ancient ties to medicine. Nevertheless, it lacks many crucial elements of more modern EM practiced in other high-income countries.

This study revealed that despite the availability of additional training opportunities in EM such as workshops and internationally accredited seminars, many physicians who work in Greek EDs have been unable to attend, citing cost, time, and concern about their ability to learn new skills at an older age. The lack of training may contribute to the reported feelings of clinical discomfort and is corroborated by the chi-squared analysis that suggests certification status is strongly associated with comfort levels. Overall, respondents were more comfortable treating critically ill patients than pediatric or trauma patients. Access to training and professional development opportunities may play a crucial role in improving comfort in treating the full breadth of ED patients.

**Figure 4:**
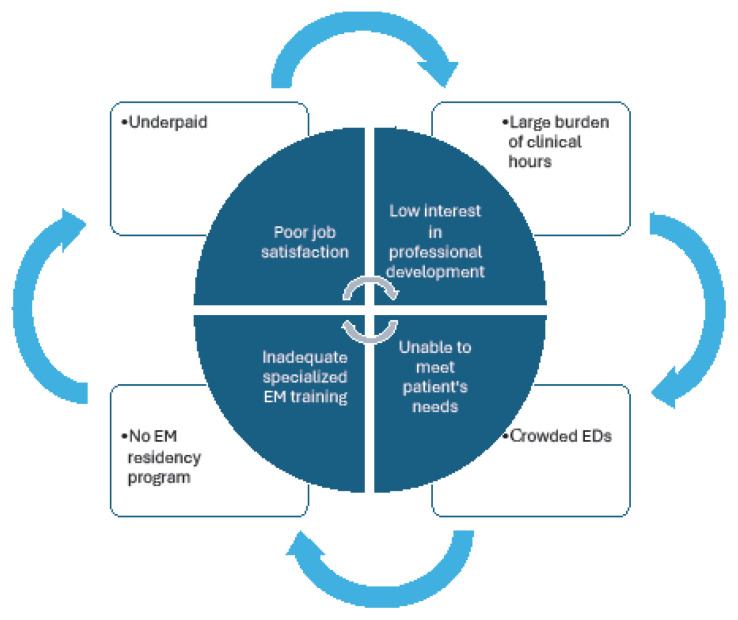
Malign cycle inhibiting Greek emergency physicians from pursuing professional development in a study of physician attitudes regarding need for emergency medicine training in Greece. *EM*, emergency medicine; *ED*, emergency department.

The study illustrates a malign cycle, depicted in Figure 4, in which Greek emergency physicians feel trapped. The lack of formal EM training and the reliance on non-EM-trained physicians may contribute to low job satisfaction, which diminishes motivation for professional development. This cycle ultimately leaves physicians inadequately prepared to meet the demands of emergency care, perpetuating a system that fails both clinicians and patients. In the background, there are other factors at play that contribute to this cycle, including low wages, high number of clinical hours, crowded EDs, and no direct path to EM from medical school (Figure 4).

The field of health promotion is not new: in 1986, the Ottawa Charter established basic strategies for health promotion advocacy and enhancing factors that promote health. In 2010, the World Health Organization (WHO) published its *Conceptual Framework for Action on Social Determinants of Health*, which stated that while societies produce both health and disease, the responsibility of promoting health equity lies with policy-makers and leaders. 8 Furthermore, it has previously been shown that the WHO framework can be used to inform the development of conceptual models that describe and visualize the key components of interventions designed to promote health.^9^ Finally, it is known that health interventions prospectively tailored to address particular barriers to healthcare are more likely to succeed.^10^,^11^

**Figure 5:**
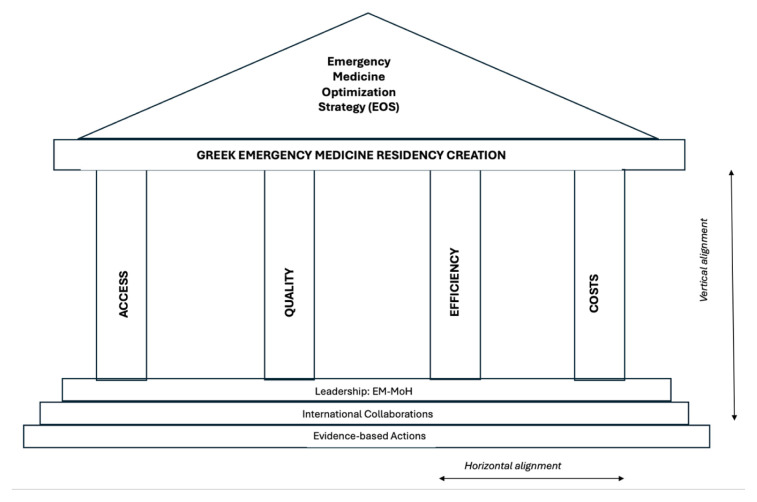
Theoretical framework for optimization of emergency medicine in Greece. *EOS*, emergency medicine optimization strategy; *EM-MoH*, Emergency Medicine-Ministry of Health.

Guided by the established frameworks of health promotion and with the goal of creating an integrated strategy to address barriers to emergency care in Greece, we developed a conceptual framework to describe a targeted strategy to improve emergency care in Greece (Figure 5).^8^

The emergency medicine optimization strategy (EOS, *Gr:Eώς*, means *“dawn”;* it is also the name of the goddess of the dawn in ancient Greek mythology) is a framework that describes how the creation of EM residency programs could improve access to care, quality of care, and efficiency of care and costs. Importantly, this requires support by a foundation of leadership at the national level, international collaborations, and research infrastructure to ensure evidence-based practices. Creating an EM residency would improve access to care across Greece’s health system by re-allocating non-EM-physicians to their clinics. When non-EM specialists staff an ED, they are often diverted from their primary duties, such as working in operating theatres, specialty clinics, and outpatient services, which leads to delays in patient care.

The quality of emergency care also suffers under this model, primarily due to insufficient training in clinical EM and, secondarily, a lack of exposure to the culture of EM. This includes essential aspects such as teamwork for managing complex, critical patients, patient advocacy, and effective communication with clinicians across specialties. The efficiency of the current system is suboptimal, due to the number of patients needing to be seen by more than one attending, as multiple consultations between specialties are common. This leads to prolonged ED evaluations and lengths of stay, resulting in crowding.

The cost of staffing Greek EDs with non-EM trained specialists is currently being studied. Preliminary data suggest that staffing Greek EDs with EM-trained physicians could reduce staffing costs by nearly 50% due to the pluripotency of emergency physicians who are able to care for the breadth of patients presenting to the ED, thus necessitating fewer consultations and providing more efficient care than non-EM trained colleagues.^12^ Furthermore, staffing hospitals in the Greek Aegean Islands with emergency physicians would likely further decrease costs due to a potential decreased need for expensive air transfers for specialty consultation from the islands to the mainland.

## LIMITATIONS

There are several study limitations. First, we did not stratify survey responses by number of years in practice, which may have masked differences arising from clinical experience. Because the qualitative results were short-answer responses, these short answers were not as in-depth compared to conducting interviews or adding focus-group interviews with a subset of study participants. Additionally, The study was disseminated by department medical directors, which may have resulted in response bias despite the anonymous nature of the survey. The observational nature of the study means that the results indicate associations rather than causation. The self-respondent nature of the survey may have resulted in sampling bias: respondents who lie within the median of emergency physicians may have been missed. Finally, the study was cross-sectional in nature and, thus, is only representative of the time during which respondents completed the survey.

## CONCLUSION

To our knowledge, this is the first assessment of the attitudes, clinical preparedness, and perceived need for EM residency training from physicians on the front lines of emergency care in Greece. Many physicians practicing in Greek EDs report being ill-prepared for the job at hand and seeing large volumes of patients. As Greece continues to take steps to improve the provision of emergency care, it will be critical to ensure that the experiences of those practicing on the frontlines are regularly heard, potentially through annual or biennial surveys, supplemented by focus-group discussions that could take place at the annual national conference. Next steps should focus on aligning actions to improve emergency care in Greece with the needs identified by physicians in the survey, including improved educational initiatives, enhancing the superspecialty training program to produce more trained EM attendings and, most critically, the creation of an EM residency training pathway in Greece.

## Supplementary Information















## Figures and Tables

**Table:** Characteristics of respondents to survey in a study of physician attitudes regarding need for emergency medicine training in Greece.

Characteristic	Number of respondents	Percent (%)
EM Certification		
Yes	40	38.1%
No	63	60.0%
Missing	2	1.9%
Primary Training Area		
Anesthesiology	5	4.8%
Cardiology	12	11.4%
General practice	17	16.2%
Orthopedics	8	7.6%
Pathology	21	20.0%
Pediatrics	4	3.8%
Pulmonology	7	6.7%
Surgery	15	14.3%
Missing	16	15.2%
Health District		
1^st^ Regional Directorate	18	17.1%
2^nd^ Regional Directorate	25	23.8%
3^rd^ Regional Directorate	7	6.7%
4^th^ Regional Directorate	28	26.7%
5^th^ Regional Directorate	10	9.5%
6^th^ Regional Directorate	13	12.4%
7^th^ Regional Directorate	1	0.1%
Missing	3	2.9%

*EM*, emergency medicine.
