# Supplementary figures and images for: Physicians in Greece’s Emergency Departments: Attitudes, Readiness, and Need for Formal Training
Source: West J Emerg Med. 2025 Jul 9;26(4):1002–7. doi: 10.5811/westjem.39964 (PMC12342500; doi:10.5811/westjem.39964)

## Greek Emergency Medicine Development Timeline

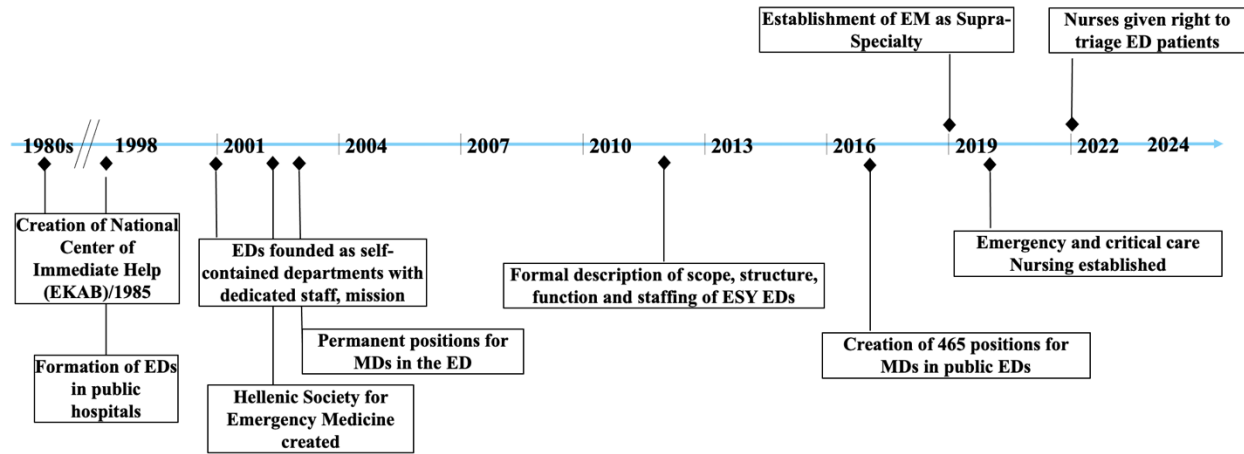

Supplement: Supplementary file 1 [file wjem-26-1002-g001.pdf]

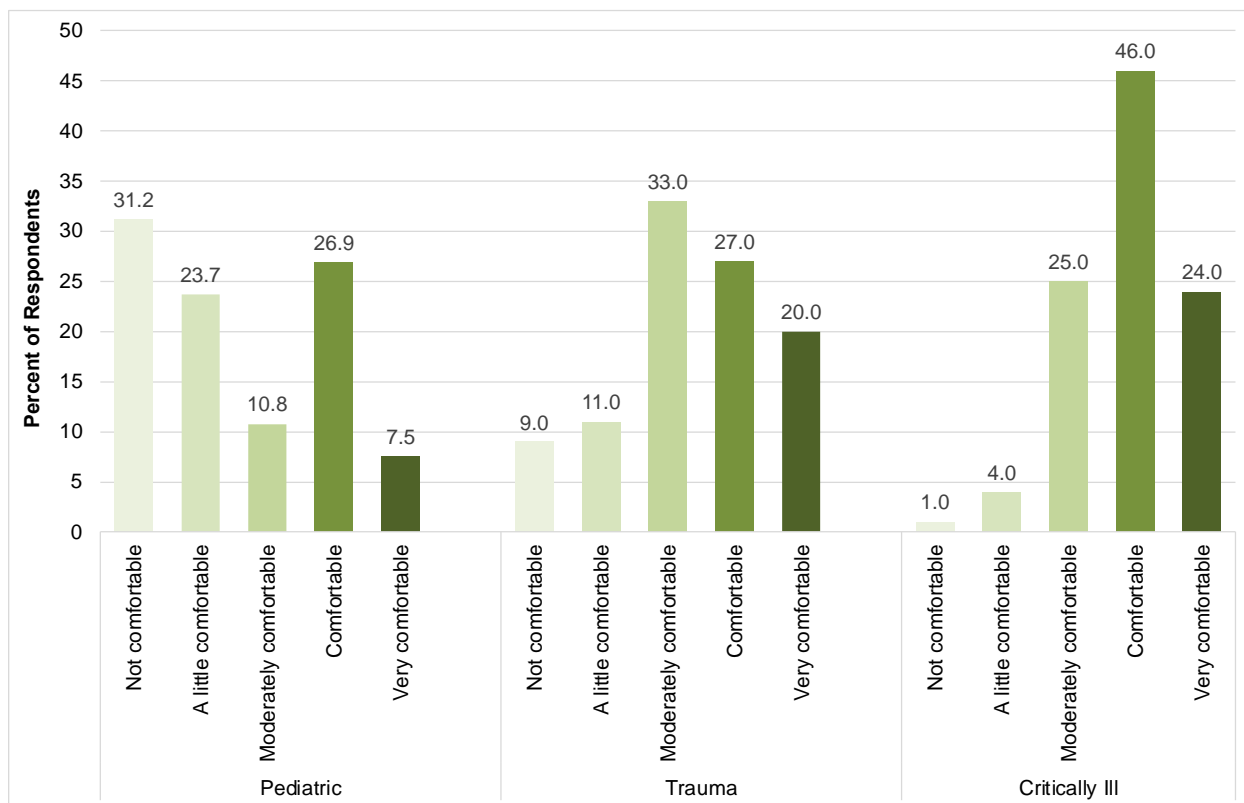

Supplement: Supplementary file 2 [file wjem-26-1002-g002.pdf]

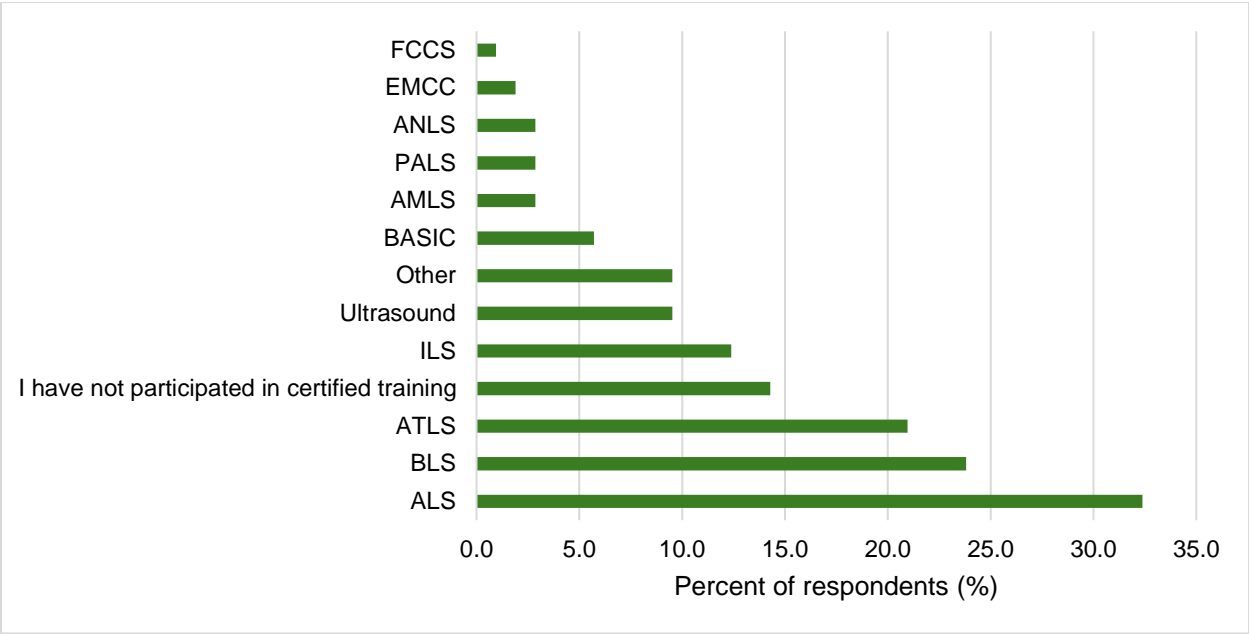

Supplement: Supplementary file 3 [file wjem-26-1002-g003.pdf]

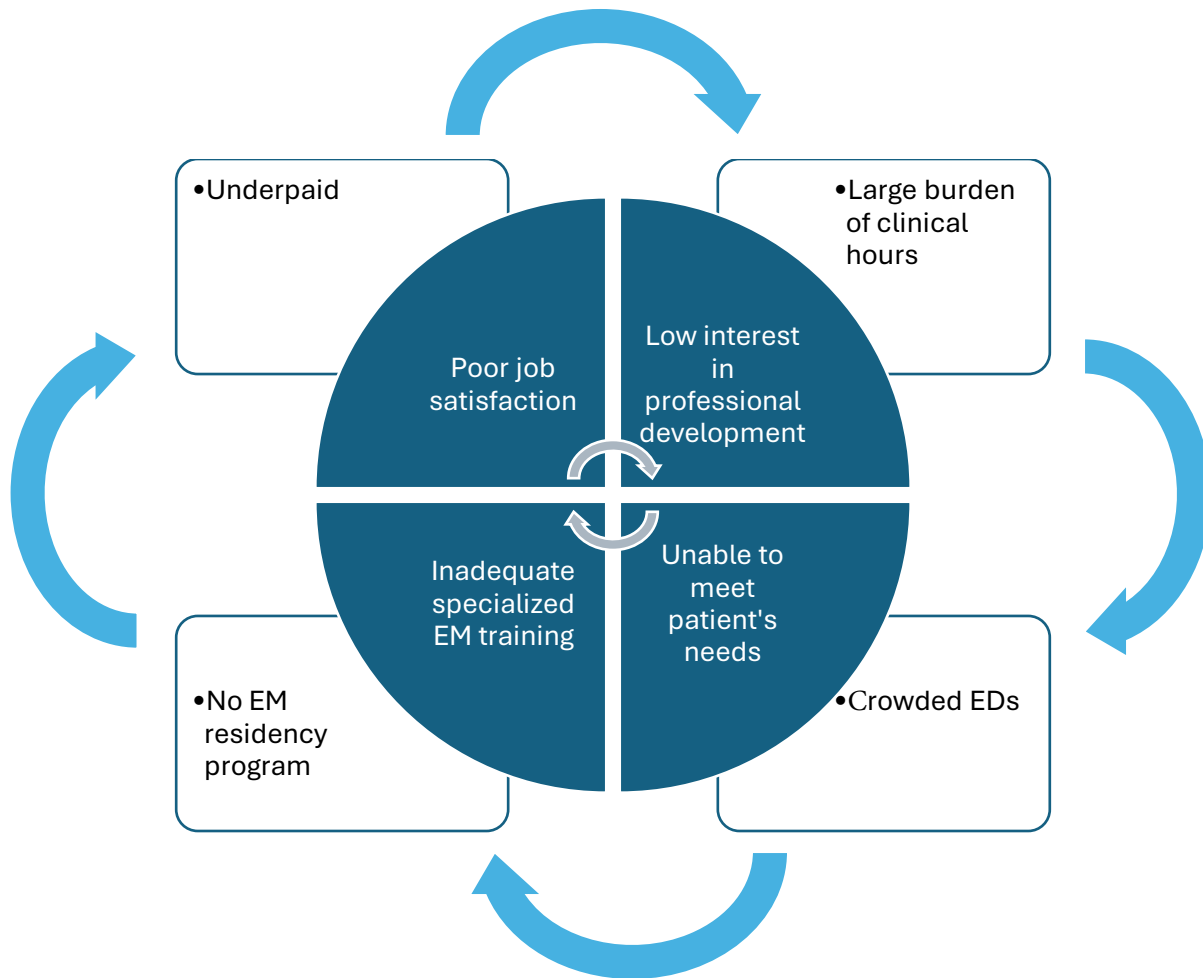

Supplement: Supplementary file 4 [file wjem-26-1002-g004.pdf]

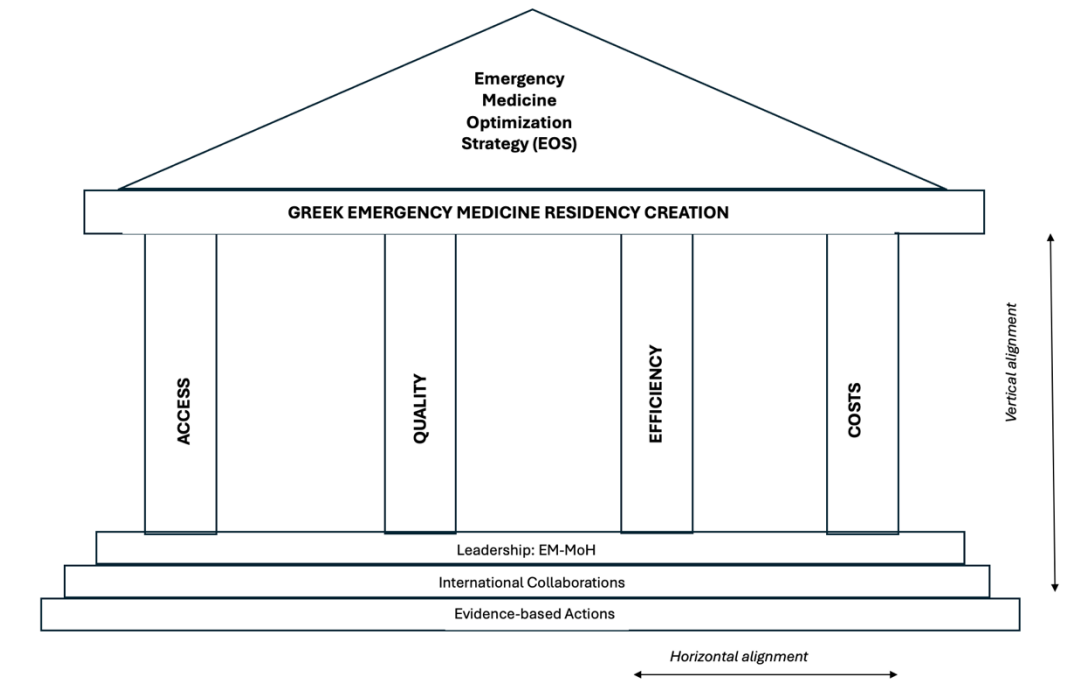

Supplement: Supplementary file 5 [file wjem-26-1002-g005.pdf]
