# Supplementary material for: Physicians in Greece’s Emergency Departments: Attitudes, Readiness, and Need for Formal Training
Source: West J Emerg Med. 2025 Jul 9;26(4):1002–7. doi: 10.5811/westjem.39964 (PMC12342500; doi:10.5811/westjem.39964)
Supplement: Supplementary file 6 [file wjem-26-1002-s001.docx]

**Appendix 1: English version of survey**

1. I have read this form, been given the chance to ask questions and have my questions answered. If I have more questions, I have been told who to contact. By selecting “I Agree” below, I am providing my signature by electronic means and agree to be in this study. I can print or save a copy of this consent information for future reference. If I do not want to be in this study, I can select “I Do Not Agree” to exit out of the survey.

I Agree

I Do Not Agree

1. Which ED do you work in?

[]

1. How is your quality of life

Bad I am overwhelmed by the workload and to tired to do anything else

Bad working in the ED is dangerous

Bad my professional expectations are not met

Fair, I have managed to balance my work and personal time

Good, it is better than working on the floor

Great I have found my calling

1. How many patients do you see per hour in a typical shift (excluding resuscitation and triage shifts)?

Less than 0.5 patient per hour

0.5-1 patient per hour

1-1.5 patient per hour

1.5-3 patient per hour

3-4.5 patient per hour

4.5-6 patient per hour

More than 6 patients per hour

1. Which of the following certifications have you taken?

ATLS

PALS

BLS

ANLS

BASIC

EMCC

AMLS

FCCS

ILS

Ultrasound

Other, *free text*

I have not taken any certifications

1. If you answered yes to the questions above, are these certifications up to date (ie not expired?)

**Would expand for each clicked off above à**

Yes

No

1. If you answered no to the question above, why are your certifications not up to date?

**Would expand for each clicked off in question 4**

Cost of certification

Have not had time

Not required for my job

I do not feel I need it

Other, *free text*

1. Are you an instructor in any of the Emergency Medicine courses?

Yes

No

1. If yes 🡪 which ones, select all that apply

ATLS

PALS

BLS

ANLS

BASIC

EMCC

AMLS

FCCS

ILS

Ultrasound

Other, *free text*

1. If no 🡪 why not?

I do not want to

I do not feel there is any benefit to being an instructor

I do not think the courses are necessary

I do not have the time to teach these courses

Other, *free text*

1. On a typical shift, what percentage of patients you are seeing are children?

None

<5 %

5-10%

10-15%

15-20%

20-25%

>25%

1. How comfortable are you with seeing children?

*Likert scale*

1. If you answered “not comfortable,” why are you not comfortable seeing children?

I do not see them enough on shift

I do not have enough knowledge about pediatrics

My training did not include pediatrics

*Other, free text*

1. On a typical shift, what percentage of patients you are seeing are trauma?

None

<5 %

5-10%

10-15%

15-20%

20-25%

>25%

1. How comfortable are you with seeing trauma?

*Likert scale*

1. If you answered “not comfortable,” why are you not comfortable seeing trauma?

I do not see enough trauma on shift

I do not have enough knowledge about trauma

My training did not include trauma

*Other, free text*

1. On a typical shift, what percentage of patients you are seeing are critically ill?

None

<5 %

5-10%

10-15%

15-20%

20-25%

>25%

1. How comfortable are you with seeing critically ill patients?

*Likert scale*

1. If you answered “not comfortable,” why are you not comfortable seeing critically ill patients?

I do not see enough critically ill patients on shift

I do not have enough knowledge about critical care

My training did not include critical care

*Other, free text*

1. On a typical shift, what percentage of patients you are seeing do you seek specialist consultation for?

None

<5 %

5-10%

10-15%

15-20%

20-25%

>25%

1. Why do you typically seek specialist consultation? (if anything other than None is selected)

*To discharge*

*To admit*

*To diagnose*

*To help with differential diagnosis*

*Free text*

1. Which specialist do you most often contact? Select all that apply

*List of specialties*

1. How do you feel about working in the emergency department?

It is great but not for me

I never signed up for emergency medicine, I was hired to see emergency cases of my specialty

I need specific training to expand my skills

I am proficient in emergency medicine skills

1. Do you think formal Emergency Medicine training (ie residency or fellowship) is needed in Greece?

I do not think it is necessary

I think it is necessary as its own residency

I think it is necessary, and can be a fellowship after formal residency training in another specialty

1. How do you feel about your position in the ED?

It was a mistake, I need to get out

It was a mistake, but I will cope

It was a good choice, but I am not proficient enough

It was a good choice, and I am trying to get better

It was a good choice, and I feel proficient enough

Still confused about how I feel about my position in the ED

1. Where do you see yourself in 5 years from now?

Advancing my career in Emergency Medicine

Doing the same things I do now in the Emergency Department

Resigning from the Emergency Department for a different NHS position in my primary specialty

Resigning from the Emergency Department and the NHS to go to the private sector

Resigning from the Emergency Department to immigrate out of Greece

Resigning from the Emergency Department and the NHS to leave medicine altogether

1. Are you EM certified?

Yes

No

1. If you answered no to the above question, are you planning on being certified?

Yes

No

1. If you answered no to the above question, why not?

No time because of working hours

No time because of personal reasons

Can not learn new things in my age

I do not want the added responsibilities

Other, *free text*

1. What is your primary specialty?
2. Do you have training in intensive care?

Yes

No

1. To which Health District does the Emergency Department you serve belong?

1st Health District

2nd Health District

3rd Health District

4th Health District

5th Health District

6th Health District

7th Health District
