# Supplementary material for: Physicians in Greece’s Emergency Departments: Attitudes, Readiness, and Need for Formal Training
Source: West J Emerg Med. 2025 Jul 9;26(4):1002–7. doi: 10.5811/westjem.39964 (PMC12342500; doi:10.5811/westjem.39964)
Supplement: Supplementary file 7 [file wjem-26-1002-s002.docx]

**Appendix 2: Certifications**

This appendix provides an overview of the certifications referenced in our article. The term "Certified" in this context denotes completion of an accredited training course, rather than a fellowship. Below is a detailed description of the certifications:

1. **Ultrasound Certification**: Includes accredited training programs such as Modular Ultrasound ESTES Course by the European Society of Trauma and Emergency Surgery (ESTES), Point of Care Ultrasound (POCUS) by the European Society for Emergency Medicine, and POCUS by the Hellenic Society for Emergency Medicine.
2. **Basic Assessment and Support in Intensive Care (BASIC)**: A short international course, typically conducted over two days, covering fundamental aspects of intensive care. In Greece, this course is provided by the Hellenic Society for Critical Care. The American equivalent, in terms of content, is the FCCS (Fundamentals of Critical Care Support) course.
3. **European Resuscitation Council (ERC) Certifications**:
   - *Basic Life Support (BLS)*
   - *Immediate Life Support (ILS)*
   - *Advanced Life Support (ALS)*
   - *Pediatric Advanced Life Support (PALS)*
   - *Neonatal Life Support (NLS)*

These certifications are accredited by the European Resuscitation Council (ERC), which aims to improve survival from cardiac arrest and other life-threatening emergencies through education and training.

1. **Advanced Trauma Life Support (ATLS)**: This certification is provided by the American College of Surgeons (ACS) and is designed to equip healthcare professionals with the necessary skills to manage acute trauma cases effectively.
2. **National Association of Emergency Medical Technicians (NAEMT) Certifications**:
   - *Prehospital Trauma Life Support (PHTLS)*
   - *Advanced Medical Life Support (AMLS)*

These internationally recognized courses focus on prehospital emergency medical care and are widely adopted by emergency medical services worldwide.

1. **Emergency Medicine Core Competencies (EMCC)**: This is an intensive simulation-based 3-day training course in Emergency Medicine taught by the European Society of Emergency Medicine. This course is designed for all clinicians, especially Emergency Medicine Specialists and Residents.
2. **EPI (ΕΠΙ in Greek)**: A national two-year training program provided by the National EMS Services (EKAB) under the auspices of the Ministry of Health in Greece. This program can be considered a fellowship equivalent and is designed to train medical doctors—both pre- and post-residency—in prehospital emergency care. All certificates issued within this program are signed by the Minister of Health.
